# Dealing with diversity—blind and visually impaired ski guiding in physical education teacher education

**DOI:** 10.3389/fspor.2025.1581913

**Published:** 2025-05-27

**Authors:** Helena Sträter, Franziska Heidrich, Iris Steineck, Ann-Kathrin Lobert, Michael Pfitzner

**Affiliations:** ^1^ Institute for Sport Science, Sport Pedagogy, University of Wuppertal, Wuppertal, Germany; ^2^ Centre for Sport Science and University Sports, Department of Sport and Human Movement Science, Subunit Education and Sports Culture, University of Vienna, Vienna, Austria; ^3^ Vienna Doctoral School of Pharmaceutical, Nutritional and Sport Sciences, University of Vienna, Vienna, Austria; ^4^ Institute for Sport Science, Department of Educational and Social Sciences, University of Hildesheim, Hildesheim, Germany; ^5^ Institute for Sport Science, Department of Sport Pedagogy and Didactics, University of Duisburg-Essen, Essen, Germany

**Keywords:** visual impairment, blindness, diversity, physical education teacher education, professionalisation, ski guiding

## Abstract

A positive attitude towards diversity seems to be crucial for successful inclusion in physical education (PE). However, some previous studies point to counterproductive attitudes of PE teachers in general and towards children with blindness or visual impairments (BVI) in particular. The present study examines how a seminar focusing on BVI ski guiding influences PE teacher education (PETE) students’ attitudes towards diversity. The seminar includes the experience of guiding a fellow student, who in turn experiences simulated BVI skiing. Subsequently, students have the option to participate in a project guiding people with actual BVI. To explore the influence of the seminar on students’ attitudes, a qualitative interview study was conducted. The findings suggest that the positive experiences go beyond the specific activity of BVI ski guiding. The seminar appears to foster the development of favourable attitudes towards diversity in general. Participants noted reduced feelings of uncertainty and an increase in self-efficacy. Nevertheless, some uncertainties persist due to the unique nature of each person and situation. The results demonstrate the benefits of purposefully designed PETE seminars that enable students to interact with people with disabilities and in this case especially those with BVI. Participation in such teaching-learning concepts entails embracing new experiences, encountering different learning environments, and overall, engaging in intense and demanding work on one's own (professional) biography, which could lead to a deeper understanding of people's needs and an open-minded attitude more generally.

## 1 Introduction

The UN CRPD emphasises that no pupil should be excluded from a mainstream school due to a disability [CRPD (1)]. From the perspective of future physical education (PE) teachers, these circumstances highlight the necessity of learning how to deal with diversity in PE. It opens up a research field of how to empower future PE teachers for dealing with diversity, understood as a broad concept that encompasses various diversity categories (2), that can be interrelated and can lead to a feeling of otherness (3) but also in specific fields like PE with blind and visually impaired (BVI) children and adolescents.

People with BVI have been intensively examined in the context of PE [e.g., (4)] and elite sport (5). A broad picture of subjective reconstructions of experiences opens up, also pointing to negative experiences such as bullying by classmates, exclusionary potential or tendencies to discriminate evinced by teachers, for example by attributing a lack of motor skills to students due to their BVI [e.g., (6, 7)]. Furthermore, PE as a subject appears to amplify the differences between people with BVI and their non-visually impaired peers more than do other subjects (6, 8). As a consequence, people with BVI tend to develop a more negative self-perception in sports, which in turn decreases their levels of physical activity later in life (7, 9).

Learning to be a teacher is multidimensional in nature, with attention needing to be paid not merely to cognitive but also to embodied, affective and motivational aspects of learning, including a shift in focus from the curriculum to what becomes meaningful to the PE teacher education (PETE) students during their PETE courses (10–13). A positive attitude towards diversity as well as being open-minded are essential for successful inclusion in PE [e.g., (14)]. However, some previous studies have highlighted the prevalence of counterproductive attitudes among PE teachers (15). One main objective of PETE programmes is to cultivate an open attitude towards diversity among prospective teachers (16). Nevertheless, in our point of view this goal often conflicts with the socialisation experiences of future teachers, shaped within a sports system that predominantly emphasises homogeneity through the segregation of athletes by presented gender, age, or performance level (17, 18).

In contrast, future PE teachers are expected to teach in a variety of classes, where students are attributed with diverse dimensions of diversity, such as varying interests, or special needs related to sports and physical activity. Addressing this diversity is a critical challenge for prospective PE teachers. Higher education in PE operates within this grey area. Consequently, the role of university teaching needs to involve encouraging PETE students to critically reflect on and deconstruct their own socialisation processes, along with the experiences and attitudes formed therein. In this context, an attitude can be understood as a predisposed mental and neural condition, shaped by prior experiences, which actively guides and influences an individual's responses to related objects and situations (19). In addition to theoretical coursework, practical experiences in diverse contexts are a crucial component of professional development. Experience with people with disabilities does not directly lead to positive attitudes. According to the contact hypothesis, positive contact leads to positive attitudes (20, 21). Pettigrew and Tropp (21) argue for avoiding anxiety and excessive demands in the contact situation to develop positive affective attitudes in particular.

According to Bandura (22) direct, personal experiences are also important for developing self-efficacy which “is defined as people's beliefs about their capabilities to produce designated levels of performance that exercise influence over events that affect their lives.” (p. 71). To sum up experiences that affect professional development have been shown to positively influence attitudes and self-efficacy regarding inclusive teaching practices in PE, enhancing teachers’ perceived competence in managing PE settings (23–26).

In Germany, PETE is organised federally and differs depending on the federal state (or *Land*). In principle, PETE is divided into two phases. The two-stage university education, which concludes with a Master of Education, and a preparatory service, which leads to a professional qualification. The university programme includes theoretical coursework as well as practical courses and internships to support professional development. With regard to diversity, the Standing Conference of Ministers of Education and Cultural Affairs in the Federal Republic of Germany (KMK) calls for a university-wide discourse on adapting the curricula of PETE programmes, including in the subject areas, as well as the networking of universities with different cooperation partners or non-school fields (27). All those involved are called upon to fulfil their responsibility to prepare future teachers to teach in heterogeneous classes.

In general, university studies in PE should influence the construction or reconstruction of perspectives on PE teaching and learning (28). It is important to consider the dual functions of PE lessons as outlined in the German curriculum (29). Firstly, the knowledge and skills acquired in PE lessons should extend beyond the classroom, promoting transferability to and practical integration in everyday life. Secondly, PE should contribute to the development of the individual's personality. Accordingly, a key aim in PETE is to foster a teaching style that is not only performance-focused but also competence-oriented (28). While a deep interest in sports and the ability to navigate performance-based assessments are essential in PETE, PE university programmes also offer opportunities to create new experiences. These experiences can encourage openness to diversity and broaden PETE students' perspectives, aligning with inclusivity-oriented and holistic educational goals.

This task was fulfilled in this project. As part of an outdoor excursion integrated into the curriculum, the programme cooperated with an association for the training of ski guides for BVI skiers. Ski guiding seems suitable insofar as the skills of a guide are important prerequisites for being able to work with people with disabilities, and an outdoor setting comes very close to the requirements profile of a teacher. In addition, the athlete-guide partnership in BVI ski guiding offers the potential for prospective PE teachers to reflect on their “[..] normative notions of ability and athleticism” [(30), p. 143]. Reconstructing these concepts through personal experience as a guide and as a BVI person offers potential to positively influence attitudes towards diversity in PE. The present study explores the impact of a seminar on BVI ski guiding on future PE teachers and the attitudes they articulated about dealing with diversity. The seminar included the experience of guiding a fellow student, who in turn experienced simulated BVI skiing. Subsequently, students were offered the opportunity to engage in a project that involves guiding people with actual BVI.

**Figure 1:**
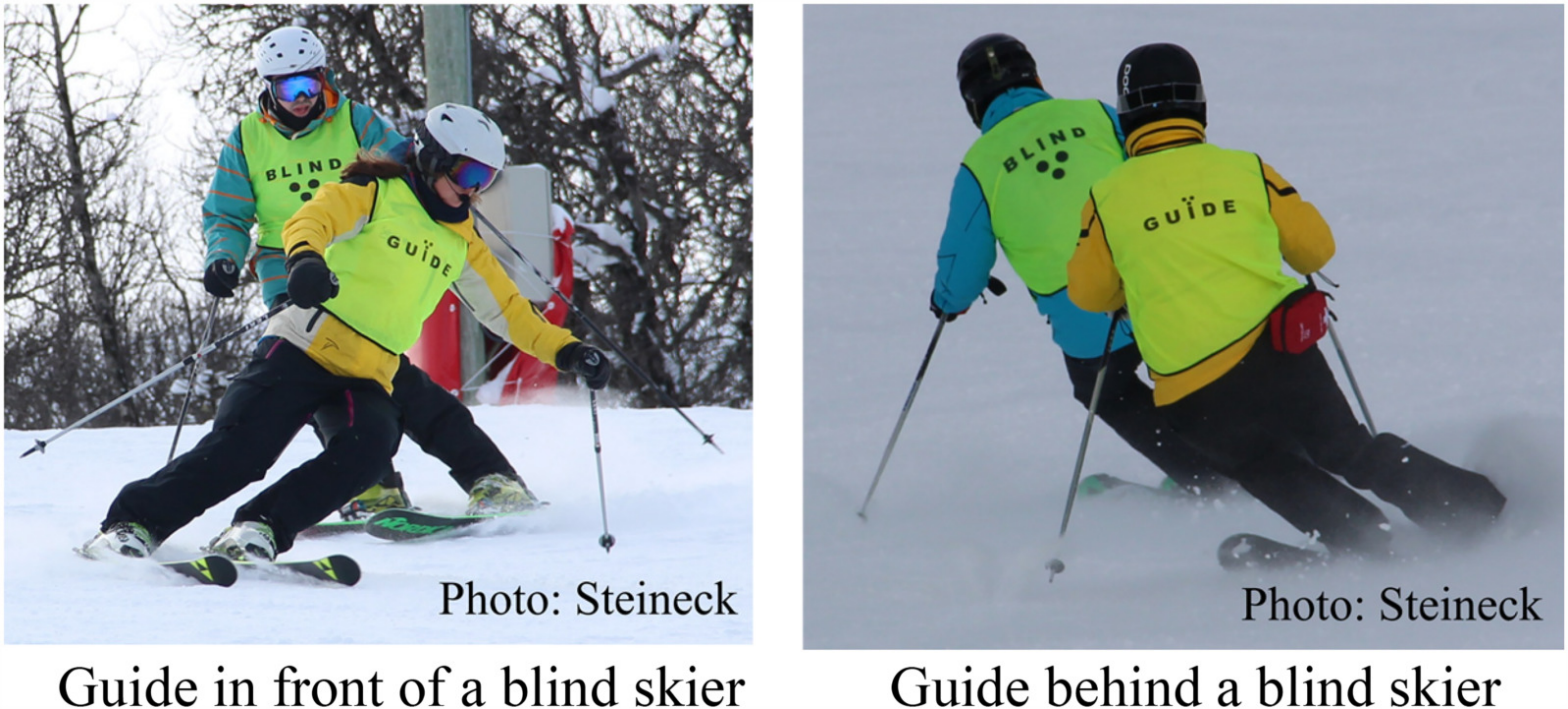
Position of the guide. © Iris Steineck.

Alpine skiing offers people with BVI a wide range of opportunities to orient themselves on the ski slope. They receive tactile and kinaesthetic information about their position in relation to the slope, the curve angles and the surface structure through the constantly changing and specifically structured acceleration and edge pressure forces (31). However, this orientation-generating information is tied to the immediate surrounding space. To ensure that BVI skiers can still participate safely in public skiing, in international practice they are led by a sighted skier, the so-called guide (32). The commands and frequency are defined in advance so that they can guide BVI skiers safely through other skiers, even at higher speeds and on full slopes (33). In popular sports, there are different ways of positioning the guide in relation to the BVI skier and different ways of using technical devices (like loudspeakers or headsets). Besides this, positioning and technical devices also depend on the preferences of the BVI person. In addition to tactile solutions, which are used for example for long inclined runs or heavily frequented passages without a significant slope gradient, the most common variants are with the guide skiing behind or in front of the BVI skier without tactile assistance (see Figure 1).

It is essential that the guide has a high level of skiing ability so that they can safely lead the BVI skiers down the slope. Furthermore their movements on the ski should be so routine and automated that they can concentrate fully on guiding and their surroundings. It is also crucial that the guide has a higher level of skiing ability than the BVI skiers so that they are not restricted in their speed and flow (34).

From other sports, such as running and tandem cycling, we know that trust, communication and responsibility are essential factors for well-coordinated guiding (35, 36). The same applies to alpine skiing. Therefore, in addition to a high level of skiing ability, strong empathy and a great sense of responsibility are essential prerequisites for a good guide (32). This is because both the guides and the BVI skiers must coordinate their needs and consider each other at all times. In addition, the guides must be willing to accept and take on the responsibility of guiding, while the BVI skiers must be willing to place their trust in the guides. For this reason, there are training courses for guides that cover not only the correct guiding techniques but also the gradual process of assuming responsibility. Furthermore, the fact that BVI skiers practice alpine skiing together with a guide transforms this sport—originally seen as an individual sport—into a team event that is “dependent on communication, trust and rapport” [(30), p. 143]. Developing a sense of responsibility and the ability to communicate seems essential in preparing PETE students to work with people with BVI and respond to their needs.

To sum up, it is long been argued that, counter to the common goal of the UN CRPD, there are proven tendencies of discrimination against people with BVI by teachers due to the (lack of) attribution of motor skills [e.g., (37)] as well as the students' negative basic attitudes towards sport and movement resulting from these negative experiences (7). In the project of BVI ski guiding investigated here, prospective PE teachers can use their own high skiing abilities and motor skills to help them serve as guides as well as being assistants in general for people with disabilities. This provides an opportunity to create an experiential space in which prospective PE teachers can engage with and experience the abilities of children, young people and adults with BVI. They also have the opportunity to broaden their perspective on PE from a primarily performance-oriented view to a more participatory one.

Such learning contexts could open up attitudes towards diversity in general. The previous considerations lead to the following research question: How do (extra-)university experiences of PETE students in BVI ski guiding influence their articulated attitudes when it comes to dealing with diversity?

## 2 Material and methods

The following section outlines the materials and methods. First the teaching-learning concept of the seminar is explained, followed by a detailed description of the study design, including data collection and data analysis.

### 2.1 Teaching-learning concept

The teaching-learning concept of the seminar focuses on contact experiences in the context of BVI ski guiding and is designed to foster the development of diversity-specific attitudes among PETE students. The seminar is an elective within the PETE program, meaning it is part of a pool of courses from which students are required to choose one. In this sense, it is not a mandatory part of the curriculum and is therefore not attended by all PETE students. The teaching-learning concept of the seminar is divided into two phases: the first phase emphasises training, while the second phase focuses on applying the acquired skills in a real-world setting. Both phases are designed to provide PETE students with new experiences in the context of diversity, using the specific example of BVI ski guiding. In the first phase, all PETE students participating in the seminar take part in a training course on BVI ski guiding, conducted in collaboration with the external partner SV Sportsgeist e.V. During the training, students alternate between the roles of guide and person with BVI, facilitating a shift in perspective. This dual role-playing allows students to experience BVI ski guiding both as the sighted person responsible for guiding and ensuring the safety of the partner, and as a person with simulated BVI (achieved through special glasses which for example simulate having blind spots/scotoma) who must rely entirely on the guide while skiing. After completing the training course, students can voluntarily participate in the Snow & Eyes project organised by SV Sportsgeist e.V. In this project, students take on the role of a ski guide for an entire week, paired with a tandem partner who is either blind or visually impaired. This second phase takes the guiding experience beyond the protected environment of the training into a real-world setting. To provide students with greater confidence in guiding, the Snow & Eyes project is framed by regular guide meetings and continuous support from designated contact persons of SV Sportsgeist e.V. These guide meetings also provided a stimulus for reflection. As part of the study, the first phase took place during the winter semester of 2021/22, and the second phase occurred in March 2023.

### 2.2 Study design

**Figure 2:**
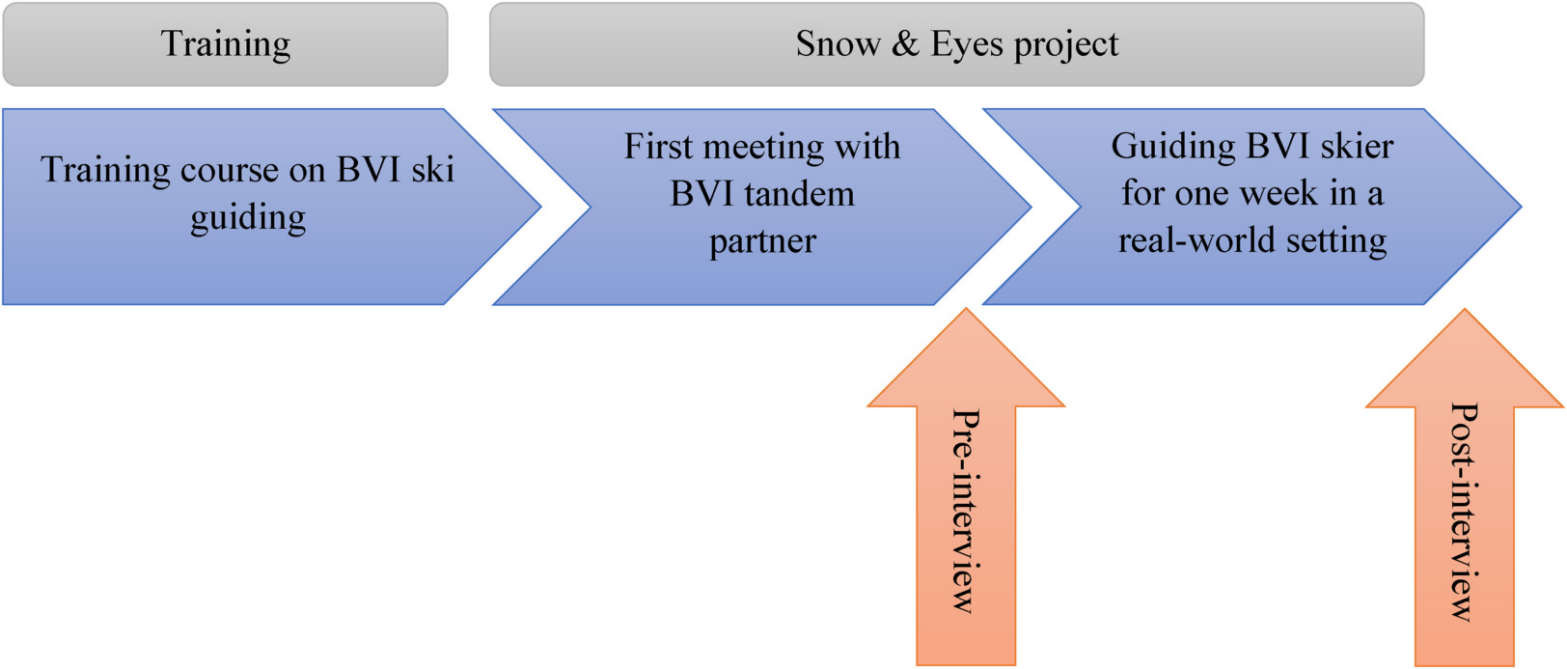
Pre-post study.

To explore the influence of the seminar on PETE students' attitudes towards diversity, a qualitative interview study was conducted. In an initial study, two students were interviewed once after participating in the Snow & Eyes project using a semi-structured interview guide. Building on this, the study design and interview guide were adjusted. In the main study, a pre-post design was applied. Students were interviewed after the first meeting with their tandem partner but before the guiding experience within the Snow & Eyes project (pre), and again approximately six weeks after the participation in the Snow & Eyes project (post), as shown in Figure 2. All pre-interviews were conducted in person. Of the post-interviews, one was conducted in person, while the remaining two were held via Zoom due to organizational constraints. The pre-interviews lasted an average of 17 min, whereas the post-interviews had an average duration of 26 min. Written informed consent was obtained from all participants prior to the start of the interviews. Semi-structured interview guides were developed based on the study's research interest and the theoretical framework. Both interviews focused on the training course and the guiding experience, with the questions aiming to encourage reflection on the participants' experiences. The interview guidelines included questions such as: What motivated you to choose the seminar? What are you looking forward to in guiding? Is there anything that makes you feel uncertain? How did you experience the guiding? Are any uncertainties still remaining? A necessary sampling criterion for the interview study was participation in both the training course and the Snow & Eyes project. Due to this specific requirement, only three students met the sampling criteria as participants. The interviews were recorded and transcribed verbatim, and then analysed by qualitative content analysis (38). Based on the research focus and the interview guides, a category system was initially developed deductively, and then tested and further refined using the data material. The category system comprises three main categories: *reasons for participation, expectations/prior attitudes*, and *attitude development*. The two categories *expectations/prior attitudes* and *attitude development* were derived deductively and are of prime importance for the results. These categories are further subdivided into the subcategories *positive expectations/prior attitudes* and *positive attitude development*, as well as *prior uncertainties* and *persistent uncertainties*. The main category, *reasons for participation*, with its subcategories *reasons for selecting/attending the seminar* and *reasons for participating in the Snow & Eyes project*, is intended to provide a clearer contextualising of the results. The category system, including descriptions, is presented in Table 1. The data were coded consensually according to the category system by two independent researchers. Disagreements between coders were discussed in joint sessions until consensus was reached. The analysis was conducted using MAXQDA software.

**Table 1:** Category system.

Category Level 1	Category Level 2	Description
Reasons for participation	Reasons for selecting/attending the seminar	Any statements in which the students provide reasons for choosing the seminar, which is not a mandatory course in PETE.
	Reasons for participating in the Snow & Eyes project	Any statements in which the students explain their motivation for participating in the Snow & Eyes project.
Expectations/prior attitudes	Positive expectations/prior attitudes	Any statements by the students that reflect positive expectations or positive attitudes prior to the Snow & Eyes project.
	Prior uncertainties	Any statements by the students that reflect uncertainties prior to the Snow & Eyes project.
Attitude development	Positive attitude development	Any statements by the students that reflect a positive attitude development concerning BVI ski guiding or inclusion or diversity in general due to the Snow & Eyes project or the seminar in general.
	Persistent uncertainties	Any statements by the students that reflect remaining uncertainties concerning BVI ski guiding or inclusion or diversity in general after the Snow & Eyes project.

## 3 Results

The following results are presented along the main categories *reasons for participation*, *expectations/prior attitudes*, and *attitude development*. Each section is illustrated with representative quotes from the interviewed students to support and contextualize the findings.

### 3.1 Reasons for participation

The findings reveal that, from the students' perspective, personal interest in inclusion was a significant criterion for selecting the seminar. One participant explains her interest in inclusion as follows:

“I’m a track and field coach and have always had a boy with Down's Syndrome in my group. I just think it's great that they can participate in these things too, and that it should become something completely normal.” (S&E Pre 01, 18)

Accordingly, it can be posited that the participants already had a fundamental openness towards inclusion. However, openness towards inclusion and a desire to learn more about it were not decisive factors for all participants when choosing the seminar. One student explained their decision to enroll as follows:

“That's a difficult question because, to be honest, I chose the seminar mainly because the timing suited me well. I didn't really know what it was about and hadn't looked into it beforehand. So I didn't have any specific motivation in that regard. But I quickly realized that the topic is actually very important for my future profession, because you’re confronted with it again and again. The seminar really showed me what you can take away from it and how you can apply it in a school context.” (S&E Pre 03, 26)

In this case, the topic of the seminar did not play any role in the participant's decision to take part—it was solely the convenient timing. Nevertheless, the student later expressed interest in the topic and recognized its professional relevance. None of the interviewed students reported being disinterested in inclusion, lacking openness toward the topic, or considering it unimportant.

Participation in the Snow & Eyes project can primarily be attributed to the students' positive experiences during the initial training course as part of the seminar. As one student put it:

“Yeah, without the seminar, I would’ve never done it. I wouldn't have gone through the training either. I just think the seminar was really well structured and kind of fun.” (S&E Pre 02, 62)

In addition to the positive experiences during the training course, further reasons mentioned for participating as a guide in the Snow & Eyes project include the desire to gain new experiences, to apply the previously acquired skills, and to support a project they perceived as meaningful and worthwhile:

“Well, because I did the training and found it really fascinating, and in my last ski vacation, I saw a tandem pair again. I thought to myself, I don't want to have done the training for nothing, I really want to try it out now. I just found it really exciting, and I’m super curious.” (S&E Pre 01, 68–70)

### 3.2 Expectations and prior attitudes

In line with the reasons outlined above—particularly the positive experiences during the training course as a key criterion for the participation in the Snow & Eyes project, and the openness and positive attitudes toward inclusion expressed in participants' reflections—the students formulated several positive expectations in advance and conveyed a generally positive and open attitude in the pre-interviews. One student expressed these expectations as follows:

“I’m really looking forward to it. I’m super curious and really excited to see how the guiding will go […] What I’m most excited about is the sense of togetherness—being able to make skiing possible for a blind person and just having a lot of fun together, sharing the experience. And also the exchange, and skiing in Norway, since I’ve never been there. But above all, I think it's about sharing those moments of success—like when you manage to make some really nice turns together.” (S&E Pre 01, 56–58)

Positive expectations included anticipation of the shared skiing experience, the expected enjoyment, and the opportunities for interaction. Moreover, the students demonstrated a sense of self-efficacy regarding their ability to guide; they felt confident in their capacity to take on this responsibility, as reflected in the following quote:

“The confidence comes from the fact that I’ve been skiing since I was a child, so I feel at least secure in that regard. During the training course, I also realized that it was actually going really well, and I felt more and more confident. In the end, it was just a lot of fun, and I no longer had that uncertainty on the slopes, so I feel confident enough to do it.” (S&E Pre 03, 80)

However, uncertainties were also evident in the students' interviews. These concerns revolved, on the one hand, around uncertainties specifically related to the act of guiding individuals with BVI in the ski area, such as recalling knowledge from the training course, managing the responsibility for another person's safety, and related fears or worries about potential mishaps. Above all, the great responsibility for another person appeared to be a source of anxiety for the students:

“I’d say I’m pretty nervous, I have some fears and concerns that something might happen, because you really do have a lot of responsibility for the other person.” (S&E Pre 02, 56)

Nevertheless, these uncertainties regarding the guiding conclude on a positive tone, with the students conveying confidence that everything will work out, as evidenced by the following statement:

“I have already thought a lot beforehand, and I am [..] especially really nervous [..] when it comes to guiding for the first time, but I actually had a really good feeling [..] while guiding [..] and felt really comfortable there, so I’m not even afraid that I won't manage it or anything, I’m just nervous because it's simply a new experience and it's the first time that I really guide someone through a ski resort and have the responsibility for someone solely on me. That's why there's a bit of nervousness, but I think once I get into it and find my flow, especially with my tandem partner, then I believe everything will go well.” (S&E Pre 03, 68)

On the other hand, students also expressed uncertainties regarding their general interaction with individuals with BVI, reflecting concerns beyond the specific aspects of skiing and guiding:

“Beforehand, we were all sitting on the train, thinking, ‘What can we ask them, and what might not be appropriate?’ You really realize that you get a bit anxious and sometimes aren't sure how to approach them.” (S&E Pre 01, 46)

### 3.3 Attitude development

The cautiously optimistic tone evident in the students' earlier expectations and uncertainties continued and was reaffirmed in their attitude development. The development of attitudes described in the interviews highlights that students were able to gain many positive experiences, contributing to a substantial shift in their perspectives. In the pre-interviews, uncertainties were more prominent, with students expressing a range of concerns about their abilities and responsibilities. Positive aspects, while present, were less frequently emphasised at this stage. After the seminar and the guiding experience, the interviews reflected a clear shift toward a more positive outlook. Positive aspects, such as increased empathy and ability to take another's perspective, a greater openness toward inclusion, a stronger sense of self-efficacy, and reduced uncertainties and fears, were far more prominent, while uncertainties and concerns were mentioned much less frequently. One student described it as follows:

“I think the uncertainty disappeared completely after the very first day and was really unfounded. Of course, there was a bit of nervousness—partly about the guiding itself, and partly, at least for me, about how to interact with the BVI participants. Like, what can you ask, what might not be okay, because I don't really know any blind people in my everyday life. So yeah, I was a bit unsure at first. But that feeling went away pretty much right on day one—already when we got off the boat, you could just tell how relaxed the atmosphere was. The blind participants really helped take away that nervousness too—they were super open, and you could ask anything. And the exchanges with the other guides also helped a lot. We talked every evening about how things went, if there were any problems or challenges. So yeah, all those uncertainties and barriers were kind of just cleared away. I always had a really good feeling, and I think it's actually good to be a little nervous at first—but that nervousness really didn't last long.” (S&E Post 01, 10)

Thus, uncertainties were resolved both in relation to the guiding itself and to the general interaction with individuals with BVI. The students' statements further indicate that the positive experiences extended beyond the specific setting of BVI ski guiding. For them, the successful implementation of inclusion in any context and of any inclusion criterion now appears much more realistic:

“We just had an intense experience, and I think it has changed us in a way or given us a different perspective on things.” (S&E Post 02, 64)

Nevertheless, a certain degree of uncertainty remains from the students' perspective. Since every person and situation is unique, taking on the responsibility of being a guide remains a challenge to some extent each time:

“I think uncertainties are always there. This was just one part we got to know, and I think it really depends on the type of disability. Even if I imagine having a blind person in my class, they’re not all the same; some might be able to manage on their own better than others. So I think you always have to approach it with a certain basic respect and make it dependent on the individual. A certain level of uncertainty is probably always present and, at first, it might even be okay and healthy to act a bit more cautiously.” (S&E Post 01, 40)

Both the experiences and reflective learning opportunities during the seminar and the Snow & Eyes project contributed to the positive development of attitudes. The seminar provided a smaller-scale environment to practise and experience guiding and perspective-taking, while the active guiding of someone with BVI during the project offered valuable contact experiences.

## 4 Discussion

This section discusses the findings of the study in light of the research questions and relevant literature. The structure follows the main categories: reasons for participation, prior expectations and attitudes, and attitude development. This is followed by a reflection on study limitations and the design of the teaching-learning concept of the seminar, as well as an outlook on future research. The final paragraph offers a short conclusion.

As the results show, participation in the seminar—consisting of the BVI ski guiding training course and the Snow & Eyes project—was motivated by different factors. The seminar is an elective course in the PETE program. Participation in the training course is a mandatory component of the seminar, whereas participation in the Snow & Eyes project is voluntary. As a reason for participation in the Snow & Eyes project, the training course served as a key moment for students that fostered confidence and sparked enthusiasm for the project. The decision to participate in the seminar was often motivated by a genuine interest in inclusion, but also pragmatic factors such as scheduling convenience played a role. This raises the question of whether the developed teaching-learning concept also reaches students who initially consider inclusion to be of little importance or approach the topic with less openness. This limitation is closely tied to the structure of the seminar itself. As an elective within the PETE program, students are free to choose from a range of seminars based on personal interest, motivation, or other practical considerations. It is precisely the specific focus on skiing—and the fact that skiing is not a mandatory component of the PETE curriculum at this university—that justifies the seminar's elective status. Moreover, the BVI ski guiding experience requires a certain level of skiing competence, further supporting the decision not to make it compulsory. Nevertheless, this raises an important implication for curriculum design (39). In order to prevent students with less interest or openness toward inclusion from avoiding such experiences entirely, mandatory courses could lay a foundation for open-minded attitudes. Required courses can play a crucial role in fostering awareness and openness toward diversity. Elective seminars like the seminar discussed in this paper then, can serve as in-depth, interest-based learning environments that build upon this foundation according to personal strengths and motivations. Furthermore, the concept described here aligns with the perspective of Schierz and Miethling (28), who emphasise the importance of PETE programmes that open up new ways of thinking about teaching and learning in PE. The experience of guiding enables students to use their advanced motor skills in skiing not solely for personal achievement but also to support others in their sporting interests, preferences and needs. This process can contribute to the development of new ways of assigning relevance in PE, moving beyond a purely performance- or training-oriented approach. In this sense, it fosters a didactic orientation that integrates cognitive reflection, informed decision-making, and communicative mediation (28).

The analysis of the prior expectations and attitudes reveal that students entered the project with a cautiously positive attitude and a strong willingness to learn, despite expressing a number of uncertainties. On the one hand, these uncertainties concerned the specific practice of guiding a person with BVI in an unfamiliar alpine environment. On the other hand, students also expressed insecurities about how to interact with individuals with BVI in general—particularly due to a lack of prior experience. This is especially evident in the pre-interviews, where students reflected on their nervousness about saying or doing the wrong thing, and about taking on full responsibility for another person's safety in the ski area. Nevertheless, these concerns are accompanied by confidence in their own competencies and a general openness to the new role. These mixed feelings reflect the ambiguity often experienced by (future) teachers in unfamiliar inclusive contexts (40). They also highlight the potential of structured, supportive learning environments to address such concerns productively (41).

The cautiously positive tone evident in the students' expectations and uncertainties was confirmed and reinforced over the course of the Snow & Eyes project. As the study shows, participation in such teaching-learning concepts involves embracing new experiences, encountering different learning environments, and overall, engaging in profound and demanding work on one's own (professional) biography. The results indicate that the participating PETE students have positively changed their initial attitudes, which were in some cases hesitant and/or marked by uncertainty or fear. It seems possible that tendencies toward discrimination against individuals with BVI—often based on incorrect or entirely absent attributions of motor skills [e.g., (37)]—can be counteracted. A normative perception of ability and athleticism appears to be challenged within the framework of the guide-athlete relationship within the BVI ski guiding (30). During the training course on BVI ski guiding, the PETE students take on the role of a BVI person by simulating BVI with special glasses. They immerse themselves in a personal experience and learn about the challenges of snow sports from this perspective. This experience deviates significantly from their usual perception of their own abilities and athleticism as sighted individuals. Familiar skills and personal athletic performance in skiing are directly influenced by the absence of visual information. This first-hand experience offers valuable moments for self-reflection. The students emphasised not only the opportunity for perspective-taking, increased openness, and reduced uncertainty but also a heightened sense of empathy and strengthened self-efficacy. The setting presented here has led students to consciously choose to work with people with BVI. Remarkably, the findings suggest that this specific setting, within a particular sport, appears to foster a more open attitude among students—one that extends beyond ski guiding, encouraging a broader receptiveness to diversity, including those with BVI in other contexts. This aligns with the findings of Foley et al. (24), who observed an increased sense of self-efficacy in those working with individuals with physical or intellectual disabilities after running a sports camp for adolescents with visual impairments. Further studies indicate that such experiences can enhance teachers' perceived competence in managing diverse PE settings and positively influence their attitudes and expectations of self-efficacy regarding inclusive PE (23, 25, 26).

Despite the promising findings, methodological limitations must also be acknowledged. The small sample size limits the generalisability of the results. Beyond the small sample size, it must be considered that social desirability may have influenced the students' responses, and thereby the study results. In addition, the results do not necessarily allow for conclusions regarding the long-term effects of the seminar or the future practical actions of prospective PE teachers in diverse or inclusive settings. According to Schierz and Miethling (28) there are indications that the transition from university to school life is challenging for prospective teachers. It should be examined whether the developed open attitude towards diversity is sustainable and is reflected in inclusion-orientated PE lessons. When reflecting on the teaching-learning concept of the seminar, the specific focus on BVI ski guiding comes to the fore. A seminar on BVI ski guiding cannot fully represent the complex nature of diversity as a whole. However, the evaluation results suggest that learning through a specific example may be beneficial, possibly by reducing complexity in a meaningful way. Moreover, the chosen learning setting provides an opportunity to highlight diversity both within and between individuals, even within what might initially appear to be a homogeneous group of people with BVI (42). “They’re not all the same; some might be able to manage on their own better than others. So I think you always have to [..] make it dependent on the individual.” (S&E Post 01, 40) suggests that PETE students shift their perspective to the individual. Nevertheless “some might be able to manage on their own better than others” (S&E Post 01, 40) should also be used as an example to critically examine the extent to which the focus is still on (dis-)abilities rather than preferences and interests in sport and movement.

Looking ahead, further research approaches appear warranted. The findings of this study should be verified and expanded through larger sample sizes. Additionally, it remains open whether the observed changes in attitudes toward diversity extend to other dimensions of diversity, allowing for similar effects to be demonstrated in different contexts. From a temporal perspective, a follow-up study would be valuable to determine whether the reported confidence in handling diversity persists over time. Furthermore, it should be examined whether these attitudinal changes translate into altered practical behaviour in diverse settings or whether this transfer—particularly from a snow sports context to general PE—poses further challenges.

This study represents a small but meaningful contribution to evaluating implemented practical elements to assess the effectiveness of such course offerings in teacher education. The findings highlight that intentionally designed teaching-learning concepts can be valuable and contribute to more positive attitudes towards diversity and inclusivity among students.

## Data Availability

The raw data supporting the conclusions of this article will be made available by the authors upon reasonable request.
